# Advancing precision rheumatology: applications of machine learning for rheumatoid arthritis management

**DOI:** 10.3389/fimmu.2024.1409555

**Published:** 2024-06-10

**Authors:** Yiming Shi, Mi Zhou, Cen Chang, Ping Jiang, Kai Wei, Jianan Zhao, Yu Shan, Yixin Zheng, Fuyu Zhao, Xinliang Lv, Shicheng Guo, Fubo Wang, Dongyi He

**Affiliations:** ^1^ Department of Rheumatology, Shanghai Guanghua Hospital of Integrative Medicine, Shanghai University of Traditional Chinese Medicine, Shanghai, China; ^2^ Guanghua Clinical Medical College, Shanghai University of Traditional Chinese Medicine, Shanghai, China; ^3^ Institute of Arthritis Research in Integrative Medicine, Shanghai Academy of Traditional Chinese Medicine, Shanghai, China; ^4^ Traditional Chinese Medicine Hospital of Inner Mongolia Autonomous Region, Hohhot, Inner Mongolia Autonomous Region, China; ^5^ Guangxi Key Laboratory for Genomic and Personalized Medicine, Guangxi Collaborative Innovation Center for Genomic and Personalized Medicine, Guangxi Medical University, Nanning, Guangxi, China; ^6^ Department of Urology, Affiliated Tumor Hospital of Guangxi Medical University, Guangxi Medical University, Nanning, Guangxi, China

**Keywords:** ML, rheumatoid arthritis, precision medicine, diagnosis, treatment

## Abstract

Rheumatoid arthritis (RA) is an autoimmune disease causing progressive joint damage. Early diagnosis and treatment is critical, but remains challenging due to RA complexity and heterogeneity. Machine learning (ML) techniques may enhance RA management by identifying patterns within multidimensional biomedical data to improve classification, diagnosis, and treatment predictions. In this review, we summarize the applications of ML for RA management. Emerging studies or applications have developed diagnostic and predictive models for RA that utilize a variety of data modalities, including electronic health records, imaging, and multi-omics data. High-performance supervised learning models have demonstrated an Area Under the Curve (AUC) exceeding 0.85, which is used for identifying RA patients and predicting treatment responses. Unsupervised learning has revealed potential RA subtypes. Ongoing research is integrating multimodal data with deep learning to further improve performance. However, key challenges remain regarding model overfitting, generalizability, validation in clinical settings, and interpretability. Small sample sizes and lack of diverse population testing risks overestimating model performance. Prospective studies evaluating real-world clinical utility are lacking. Enhancing model interpretability is critical for clinician acceptance. In summary, while ML shows promise for transforming RA management through earlier diagnosis and optimized treatment, larger scale multisite data, prospective clinical validation of interpretable models, and testing across diverse populations is still needed. As these gaps are addressed, ML may pave the way towards precision medicine in RA.

## 1 Introduction

Rheumatoid arthritis (RA) is a prevalent autoimmune disorder characterized by inflammation and discomfort in numerous small joints, potentially leading to joint deformity and impaired functionality. Furthermore, it ranks among the primary contributors to chronic disability (1). Furthermore, RA not only impacts the joints but also has implications for other bodily systems, including the cardiovascular and respiratory systems, leading to an elevated susceptibility to conditions such as myocardial infarction, stroke, and pulmonary fibrosis (2, 3). Chronic illnesses and persistent pain can result in psychological distress for patients, manifesting as symptoms of depression and anxiety (4). Hence, it is imperative to promptly identify individuals with a high susceptibility to RA in order to facilitate early diagnosis and anticipate the potential severity of disease progression. Furthermore, the timely administration of efficacious medications is essential in impeding the advancement of the disease.

The phrase “machine learning (ML)” surged in popularity in the late 1990s in the field of artificial intelligence (5). In the past decade, ML has made significant advancements as a result of the increased availability of data and improvements in algorithms, enabling the identification of complex patterns and correlations within datasets (6). The biomedical field has experienced a significant increase in data volume, ranging from molecular details to comprehensive information on the human body system, due to advancements in high-throughput sequencing technologies, electronic health records, and medical imaging (7). Healthcare providers and researchers are currently facing a growing number of clinical challenges, leading them to explore ways to enhance decision-making effectiveness, refine personalized treatment strategies, and optimize resource allocation methods. ML is uniquely positioned to extract valuable patterns and insights from large datasets, potentially automating and enhancing the efficiency of healthcare decision-making and services. The incremental incorporation of biomedicine with various disciplines, including computational science, mathematics, and statistics, has spurred interdisciplinary partnerships, leading to accelerated progress in the application of ML in the field of biomedicine (8). In the clinical practice of RA, Rheumatoid Factor (RF) and Anti-Citrullinated Protein Antibody (ACPA) serve as crucial diagnostic biomarkers for RA, playing key roles in its diagnosis. However, approximately 20-25% of RA patients are seronegative, posing challenges to early diagnosis and potentially leading to delayed diagnosis and treatment (9). With the advent and development of biologics, significant progress has been made in the treatment of RA. Nevertheless, many RA patients exhibit poor responses to drug treatments, failing to achieve sustained remission (10), and currently, it is not possible to predict which treatment drugs will have the best therapeutic effect on individual patients. The accumulation of biomedical big data may provide new insights into better understanding the heterogeneity of RA (11). With the increase in data volume and complexity, traditional statistical analysis methods have become insufficient, especially when dealing with nonlinear relationships and complex interactions between variables (12). These unmet needs pose challenges to the precision medicine of RA. Using ML techniques for data processing and pattern recognition to build predictive models for RA can assist clinicians in making more accurate data-driven decisions (13). Therefore, understanding the prevalent ML algorithms in RA, their effectiveness, and potential applications is crucial. Our study is dedicated to evaluating recent literature on applications of ML in RA classification and outcome prediction, with the goal of offering a dependable benchmark for reference and guiding future research endeavors. By enhancing the utilization of sophisticated modeling in RA and advocating for precision medicine in the field, our work aims to propel advancements in RA treatment and management.

## 2 ML algorithms to enhance precision rheumatology

ML, a crucial component of artificial intelligence, is divided into two main categories: supervised and unsupervised learning. Supervised learning employs labeled training datasets to identify patterns and relationships. Upon training, the model can predict or classify new data inputs, yielding corresponding results. This method utilizes a range of algorithms, such as logistic regression, random forests, gradient boosting, and decision trees. Each algorithm contributes uniquely to the robustness and accuracy of predictive outcomes, making supervised learning integral to advancements in data-driven research methodologies (14). Supervised learning is divided into two principal methodologies: classification and regression (15). Classification methodologies segregate patients according to distinct characteristics (16). By employing datasets comprising genetic information, gene expression profiles, and clinical indicators from patients with RA, algorithms can be trained to identify RA patients within populations, as well as to ascertain which patients exhibit optimal responses to specific treatments. Regression models, on the other hand, are designed to predict continuous outcomes (17), such as disease activity scores and response rates to treatments in RA patients, thus facilitating personalized monitoring and management to optimize treatment efficacy. In contrast, unsupervised learning explores inherent patterns and relationships in datasets without predetermined labels (18). Clustering algorithms, an exemplary application of unsupervised learning, automatically group data into multiple clusters to maximize intra-cluster similarity and minimize inter-cluster similarity, aiding significantly in RA research by identifying potential patient subgroups who may exhibit favorable responses to specific treatments or distinct disease progression patterns. Deep learning, employing Artificial Neural Network (ANN) technologies, enhances the analysis and prediction of complex data through sophisticated non-linear mapping relationships (19). Particularly, Convolutional Neural Networks (CNNs) in deep learning architectures are adept in processing image data (20), enabling automatic feature learning from multiple convolutional layers which assist physicians in identifying early signs of arthritis or disease progression in X-ray or Magnetic Resonance Imaging (MRI) images of RA patients. In summary, supervised and unsupervised learning each serve specific roles, while deep learning technologies enhance the capability of these methods to process complex data, thereby effectively advancing the field of precision rheumatology.

In the preprocessing phase, data cleaning and organization are paramount, involving the removal of duplicates and correction of anomalies (21). Furthermore, feature engineering plays a critical role in identifying predictors (x) that significantly influence the target variable (y) through strategic selection and transformation of data, a crucial task in supervised learning. Accurate feature selection not only enhances the precision of the model but also its interpretability. When constructing predictive models, addressing the challenge of managing a large volume of available features is commonplace. While the use of advanced and efficient algorithms is vital, ineffective predictive information derived from these features, or the presence of numerous irrelevant variables, can impair model performance. Implementing key feature selection strategies is crucial, including statistical filtering, wrapper methods, and advanced embedded techniques (22–24). For instance, Random Forest assesses feature importance by calculating their contribution to model accuracy (25), whereas Logistic Regression identifies key influencing factors by analyzing the magnitude and direction of coefficients (26). Through rigorous feature selection, the dimensionality and complexity of the dataset are effectively reduced, thereby enhancing the interpretability and practical application of the predictive model in clinical decision-making (22). For example, identifying RA patients with specific genetic mutations through feature selection has indicated that these individuals respond more positively to methotrexate, a principal drug for RA treatment. This insight assists physicians in devising targeted treatment plans, thereby improving therapeutic outcomes.

**Figure 1:**
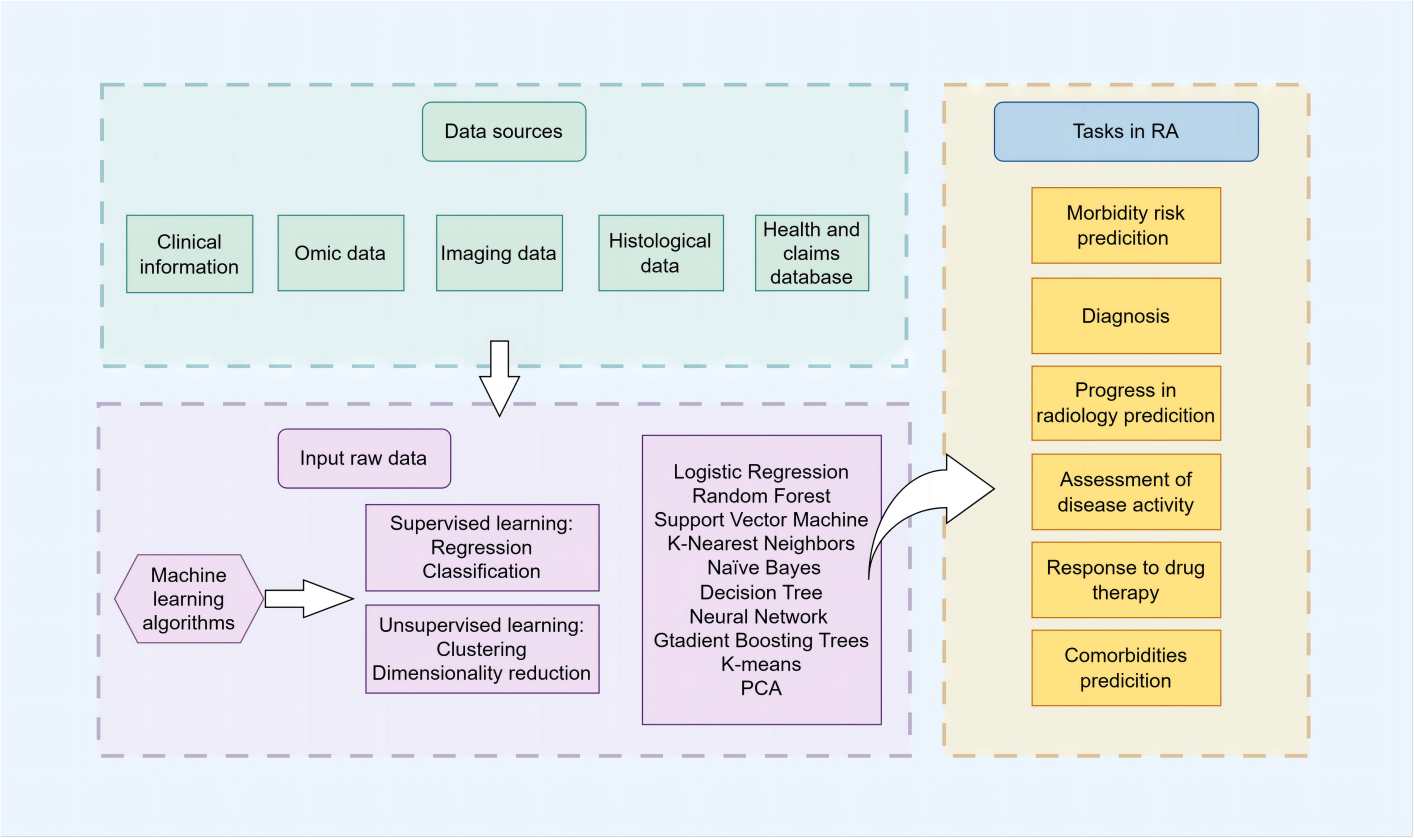
Schematic overview of clinical prediction in RA using ML The schematic illustrates the comprehensive workflow and applications of ML algorithms in the management of RA. It encapsulates the stepwise process from data collection, including electronic health records, imaging, and multi-omics data, through data preprocessing and feature engineering, to model training and validation phases. The central part of the diagram highlights the primary domains of ML application in RA: risk prediction, diagnosis and subtype classification, prediction of disease activity and progression, treatment response, and comorbidity identification for RA. It emphasizes the iterative optimization of models and the synergy between clinical and computational insights aimed at advancing early diagnosis, personalized treatments, and patient outcomes in RA management.

ML algorithms are increasingly recognized as powerful analytical tools in the field of RA research. As depicted in **Figure 1**, they provide assistance across multiple domains, including diagnosis, disease progression forecasting, prediction of treatment responses, and identification of potential complications. These computational tools are guiding the field towards a more refined and individualized approach, allowing clinicians and researchers to explore the complexities of RA with greater accuracy.

## 3 ML models in precision diagnosis and therapeutics for RA

A variety of predictive models have been built using ML algorithms in RA research. Presented in **Table 1** is the appraisal of performance when these ML models serve as classifiers across a multitude of data types from various sources. The functionalities of these classifiers include identification of individuals at risk for RA, diagnosis and differentiation of subtypes, discrimination of disease activity levels, forecasting of treatment outcomes as effective or ineffective, and predicting the presence or absence of comorbidities.

**Table 1:** Application of ML in RA.

Task	Sample Size		Features	ML algorithms	Performance	Ref
Risk Prediction	Training set: RA patients: n = 599 Controls: n = 1673 Test set 1:RA: n = 125 Controls: n = 349 Test set 2:RA: n = 127 Controls: n = 355 Test set 3:RA: n = 127 Controls: n = 355		9 SNPs	LR, SVM, Naïve Bayes, RF, XGBoost	AUC > 0.9	(27)
	RA or no arthritis: n =17,366 Training set: n = 8683 Validation set: n = 4342 Test set: n = 4341		Age, gender, race, high BMI, gout, diabetic, smoked, sleep, blood pressure, patient health questionnaire, income to poverty ratio	Bayes	validation set: AUC = 0.826 test set: AUC = 0.805	(28)
	Training cohort: RA: n=47 non-RA: n=64 Test cohort: UA: n = 62		the Leiden prediction rule, 12-gene risk metric	SVM	AUC = 0.84	(29)
	UA: n = 72, RA: n = 8, HD: n = 13		cpg sites, clinical parameters	LR, SVM, RF	AUC: 0.875-1	(30)
Diagnosis	hand radiograph images: Training set: RA: n = 256 OA: n = 262 Normal: n = 231, Others: n = 242; Validation set: RA: n = 56 OA: n = 57 Normal: n = 51 Others: n = 53; Test set: RA: n = 56 OA: n = 58 Normal: n = 51 Others: n = 53		–	CNNs	Classification of RA and normal: AUC = 0.97 Classification of RA and OA and normal: Acc = 0.806 Classification of RA and OA and normaland others: Acc = 0.844	(31)
	1337 RA ultrasound images of 208 patients		–	DL	Classification of synovial proliferation or not: Group1/Group2/Group3: AUC = 0.863/0.861/0.886 Classification of healthy and diseased: Group1/Group2/Group3: AUC=0.848/ 0.864/0.916	(32)
	Training set: HC: n = 100 RA: n = 100 Validation set: HC: n = 18 RA: n = 20		hand images, Age, gripforce	BayesNet, NaïveBayes, Logistic, k-NN, RF,etc.	Classification of RA and HC Acc = 0.947 Sen = 0.95 Spe = 0.944 AUC = 0.971	(33)
	Training set: GSE93272, GSE45291, GSE74143, GSE65010, GSE15573, GSE61635, GSE65391, GSE138458, GSE143272, GSE113469, GSE50772 Test set: GSE55457,		15 key genes	LASSO, SVM, RF,XGBoost, BPNN, CNN	AUC > 0.85	(34)
	GSE93272, GSE17755		MAPK3, ACTB, ACTG1, VAV2, PTPN6, ACTN1	LASSO	Training set: AUC= 0.801 Validation set: AUC= 0.979	(35)
	Uninflamed: n = 10 Resolving arthritis: n = 9 Early RA: n = 17 Established RA: n = 12		cytokine, chemokine	GMLVQ	RA vs. non-inflamed group: AUC = 0.996 Early RA vs. resolved arthritis group: AUC = 0.764	(36)
	Training set: GSE12021, GSE55235, GSE55457, GSE55584 Validation set: Dataset1: GSE89408 Dataset2: GSE77298, GSE153015		m6A methylation regulators	RF, Rpart, LASSO, XGBoost, LR	Classification of RA and HC AUC = 0.85 (IGF2BP3) AUC = 0.85 (YTHDC2)	(37)
	Serum of 225 RA patients and 100 HC Discovery set: n = 243 Validation set: n = 82		26 metabolites and lipids	LR, RF, SVM	Classification of RA and HC: AUC = 0.91 Sen = 0.897 Spe = 0.906	(38)
	Test cohort: RA: n=36 OA: n=18 HC: n=18 Validation cohort: RA: n=24 OA: n=12 HC: n=12		3 groups of differentially expressed proteins	RF	Classification of RA: AUC = 0.9949 Classification of ACPA-positive RA patients: AUC = 0.9913 Classification of ACPA-negative RA patients: AUC = 1.0	(39)
	IBD: n = 14, MS: n = 7, RA: n = 5, JIA: n = 3, SLE: n = 3, T1D: n = 2, BS: n = 2, AS: n = 2, APS: n = 1、PSC: n = 1, MG: n = 1, ReA: n = 1		gut microbiome	RF, SVM , XGBoost, Ridge Regression	Classification of RA and IBD: AUC > 0.86 Classification of RA and MS: AUC > 0.96	(40)
	Discovery cohort: 167 RA and 91 controls Validation cohort: 12 SLE、32 RA and 32 controls		miR-22-3p, miR-24-3p, miR-96-5p, miR-134-5p, miR-140-3p, miR-627-5p	LASSO, RF, LR	Classification of RA and non-RA: AUC = 0.71 Classification of ACPA-positive RA and others: AUC = 0.73 Classification of ACPA-negative RA and others: AUC = 0.73	(41)
	H&E-stained images of TKR explant synovium (OA: n = 147, RA: n = 60) Training set: n = 166 Test set: n = 41		14 pathologist-scored features、computer vision-quantified cell density	RF	Classification of RA and OA AUC = 0.91	(42)
	129 synovial tissue samples RA: n = 123 OA: n = 6		histologic scoring	SVM	Classification of the high inflammatory subtype and others: AUC = 0.88 Classification of the low inflammatory subtype and others: AUC = 0.71 Classification of the mixed subtype and others: AUC = 0.59	(43)
Disease activity/ imaging progression	Hanyang Bae RA Cohort: No progression: n = 118 Severe progression: n = 120 NARAC Cohort: No progression: n = 68 Severe progression: n = 86		genetic and clinical factors	SVM	Classification of radiologic progression and no progression AUC = 0.7872	(44)
	ultrasound images from RA patients Training set: n = 1678 Testing set: n = 322		–	CNN	Distinguishing class 0 from the other classes: AUC = 0.96 Distinguishing class1 from class 2 and 3 classes: AUC = 0.94 Distinguishing class 2 from class 3 classes: AUC = 0.93	(45)
	135 visits from 41 patients		dose percentage change, the DAS-28 ESR score, ESR, disease duration, CRP, and the duration of remission at study entry	LR, KNN, NB, RF, Stacking-Meta Classifier	Classification of flare yes and. flare no AUC: 0.72 - 0.81	(46)
	stable RA patients: n = 130 training set: n = 104 test set: n= 26		baseline serum proteomics	LASSO, XGBoost	Classification of flare and remission AUC = 0.8	(47)
	2 electronic health record platforms UH Cohort: n = 578 (Training Cohort : Test Cohort: n= 116) SNH Cohort: n= 242 (Training Cohort: n = 125, Test: n = 117)		medications, patient demographics, laboratories, and prior measures of disease activity.	DL	Classification of controlled and uncontrolled UH training model test in UH Test Cohort: AUC = 0.91 UH training model test in SNH test Cohort: AUC = 0.74	(48)
	300 RA patients		laboratory data, medicare claims and medications	LR	Classification of high/moderate and low disease activity/remission AUC = 0.76	(49)
	Optum dataset:n = 68,608 Externally validatiation: IBM CCAE: n = 75,579 IBM MDCD: n = 7,537 IBM MDCR: n = 36,090		health service utilization, demographics, prescription claims for immunosuppressants, steroids, DMARDs, pain medications, and other comorbid conditions.	regularized LASSO, LR, RF, GBM	90-day TAR: AUC (IBM CCAE) = 0.77, AUC (IBM MDCR) = 0.75, AUC (IBM MDCD) = 0.77, 730-day TAR: AUC = 0.71	(50)
Terapeutic response	MTX	All patients with new onset RA Training cohort: n = 26 Validation cohort: n = 21	metagenomic, clinical-­pharmacogenetic variables	RF	AUC = 0.84	(51)
		Training dataset: ESPOIR: n = 493 EAC: n = 239 External validation dataset: Treach: n = 138	DAS28, creatininemia, leucocytes, lymphocytes, AST, ALT, swollen joints count and corticosteroids co-treatment.	LR, RF, LightGBM, CatBoost	Training dataset: AUC = 0.73 External validation set: AUC = 0.72	(52)
		349 RA patients: Training set: n = 279 Test set: n = 70	95 haplotypes and 5 non-genetic factors	NN, SVM, LR, EN, RF, Boosted Trees	AUC: 0.776 - 0.828 Sen: 0.656 - 0.813 Spe: 0.684 - 0.868	(53)
		82 RA patients: good responders: n = 42 poor responders/nonresponders: n = 43	gene expression	L2-regularized LR, RF, network‐based approach	predictive utility between 4 weeks and pretreatmen: acc = 0.61, AUC = 0.78 predictive utility at the 4‐week time point: acc = 0.68, AUC = 0.78.	(54)
	TNFi	Discovery cohort: n = 74(52 responders and 22 non responders) Validation cohort: n = 25(14 responders and 11 non responders)	clinical and molecular parameters	LR	AUC = 0.91	(55)
		Training dataset: n = 1892 Testing dataset: n = 680	demographic, clinical, and genetic markers	linear models, CART, SVM, GPR	Training dataset: AUC = 0.66 Testing dataset: AUC = 0.615	(56)
		Synovial tissue samples: RA: n = 256, OA: n = 41 NC: n = 36; Genes: n = 11,769	pathway and DEG	NB, DT, KNN, SVM	For infliximab response: Pathway-driven model AUC = 0.87, AUPR = 0.78; DEG-driven mode AUC = 0.92, AUPR = 0.86	(57)
		179 RA patients: Training set: n = 141 Validation set: n = 38	9 clinical parameters	NN	Response to infliximab AUC = 0.75	(58)
		responders: n = 23 non-responders: n = 16	clinical data, flow cytometry measurements, protein measurements and transcriptomics data	Linear, non-linear, kernel-based	response to TNFi AUC = 0.81	(59)
		Training set: n = 161 Validation set: n = 118	DAS28, lymphocytes, ALT, neutrophils, Age, weight and ever smoked	LR, RF, XGBoost, CatBoost	Response to Etanercept: Training set: AUC = 0.74 Validation set: AUC = 0.70 Response to monoclonal anti-TNF antibodies: Training set: AUC = 0.74 Validation set: AUC = 0.71	(60)
	Other drugs	R4RA synovial biopsies: n = 164	gene expression, clinical data and histological data	elastic net regression, GBM	For rituximab response AUC = 0.744 For tocilizumab response AUC = 0.681 For refractory state: AUC = 0.686	(61)
		1204 patients treated with bDMARDs	age, rheumatoid factor, ESR, disease duration, CRP	Lasso, Ridge, SVM , RF, XGBoost	Acc: 0.528 - 0.729 AUC: 0.511 - 0.694	(62)
		Training set: n = 625 Independent test set: n = 322	PtGA	RF, XGBoost, ANN, SVM	Acc = 0.726 AUC = 0.638 F1 score = 0.841	(63)
		Training set: 51 MR and 85 NR External validation cohort: 35 MR and 47 NR	DAS-28	CART	Training set: AUC = 0.89 Sen = 0.88 Spe = 0.94 Validation cohort: AUC = 0.82	(64)
Comorbidities	487 patients diagnosed with RA and osteoporosis Training set: n = 340 Testing set: n = 147		baseline demographic, clinical test indicators	RF, ANN, SVM, XGBoost, DT	Training set: AUC = 0.878 Testing set: AUC = 0.872	(65)
	2374 RA patients		clinical features, medication, laboratory results	LR, RF, XGBoost, LightGBM	AUC = 0.75 Acc =0.68 F1 score = 0.7	(66)
	2 atherosclerosis and 2 RA datasets		NFIL3, EED, GRK2, MAP3K11, RMI1, TPST1	LASSO, RF	AUC: 0.723 to 1	(67)
	Training cohort: RA+CHD: n = 294 RA: n = 718 Validation cohort: RA+CHD: n = 70 RA: n = 204		age, hypertension, anti-CCP antibody positivity, rheumatoid factor positivity, a high ESR, high CRP levels, and dyslipidemia of LDL-c, TC, triglycerides and HDL-c	GBDT, KNN, LR, RF, XGBoost, SVM	AUC = 0.77 Sen = 0.639 Spe = 0.772	(68)
	RA-ILD: n = 75 RA-non-ILD: n = 78		age, KL-6, D-dimer, CA19-9	LASSO, RF, PLS	AUC = 0.928 Sen = 0.83 Spe = 0.81	(69)

Acc, accuracy; ADA, adaptive boosting; ALT, alanine aminotransferase; AST, aspartate aminotransferase; APS, antiphospholipid syndrome; AS, ankylosing spondylitis; AUPR, area under the precision-recall; BMI, body mass index; BS, behcet’s syndrome; b/tsDMARDs, biologic or targeted synthetic disease modifying antirheumatic drugs; CART, classification and regression tree; CA19-9,carbohydrate antigen 19-9; CCP, cyclic citrullinated peptide; CHD, coronary heart disease; CRP, c-reactive protein; DAS 28, disease activity score-28; DEG, differentially expressed gene; DL, deep learning; DT, decision tree; EN, elastic nets; ESR, erythrocyte sedimentation rate; GBDT, gradient boosting decision tree; GBM, gradient-boosted machine; GPR, gaussian process regression; HC, healthy control; HDL, high density lipoprotein; IBD, inflammatory bowel disease; ILD, interstitial lung disease; JIA, juvenile idiopathic arthritis; KL-6, Krebs von den Lungen-6; KNN, k-nearest-neighbors; LASSO, least absolute shrinkage and selection operator; LDL, low density lipoprotein; LR, logistic regression; MG, myasthenia gravis; MR, multi-refractory; MS, multiple sclerosis; MTX, methotrexate; Non-ILD, rheumatoid arthritis-without interstitial lung disease; NB, naïve bayes; NN, neural networks; NR, non-refractory; OA, osteoarthritis; OP,osteoporosis; PLS, partial least square; PRS, polygenic risk score; PSC, primary sclerosing cholangitis; PtGA, patient global assessment of disease activity; R, responders; RA, rheumatoid arthritis; ReA, reactive arthritis; RF, random forest; SEN, sensitivity; SLE, systemic lupus erythematosus; SNH, safety-net hospital cohort; SNP, single nucleotide polymorphism; SPE, specificity; SVM, support vector machine; TAR, time at risk; TC, total cholesterol; T1D, type 1 diabetes; TNFi, tumor necrosis factor inhibitor; TKR, total knee replacement; UH, university hospital cohort; XGBoost, eXtreme Gradient Boosting.

### 3.1 Stratification of RA risk cohorts

Identifying individuals at risk for RA is crucial for early intervention, which has been shown to yield substantially better outcomes when applied during the preclinical stages rather than after the overt development of clinically significant arthritis (70). Specifically, by identifying individuals at high risk and conducting regular medical examinations and monitoring RA-related biomarkers, such as inflammation levels and autoantibodies, early detection of the disease can utilize the ‘window of opportunity’ for therapeutic intervention. Early interventions can help prevent severe radiographic damage and disability, thus significantly improving patient prognosis (71). The exact etiology of RA remains not fully understood; however, it is known that genetic and environmental factors, as well as their interactions, influence the onset and progression of RA (72). ML, as an effective data analysis tool, is capable of processing and interpreting large volumes of diverse data, ranging from genetic factors to lifestyle choices. ML can uncover potential risk patterns within complex genetic and environmental datasets, assisting clinicians in making more accurate disease predictions and risk assessments.

Predictive modeling harnessing ML techniques to pinpoint individuals at an elevated risk for RA can be principally segregated into two domains: forecasting the incident risk in asymptomatic persons and assessing the progression likelihood in symptomatic patients with undifferentiated arthritis towards RA. The detection of RA susceptibility in the broad population leans on the analysis of genetic variants alongside common clinical risk indicators such as family history, age, and gender. A study found nine single nucleotide polymorphisms (SNPs) linked to RA, by combining these variations into a risk score and using ML algorithms, researchers were able to accurately distinguish RA patients from those without the condition, exhibiting five-fold cross-validated AUCs surpassing the 0.9 threshold (27). 11 risk factors for RA were identified from National Health and Nutrition Examination Survey (NHANES) data and used to create a Bayesian logistic regression model, which was refined using a Genetic Algorithm. The model showed high predictive accuracy with an AUC of 0.826 on the validation set (28). These findings highlight the potential of machine learning strategies in predicting risk populations for RA. Genetic risk scores derived from SNPs can help identify an individual’s potential genetic risks, thereby providing a crucial foundation for personalized medicine (73). However, translating these studies into clinical decision support tools faces obstacles, primarily ensuring the equal applicability of Polygenic risk score (PRS) across populations (74). In reality, PRS exhibits limited transferability among populations, and its clinical utility in RA remains undetermined, necessitating substantial investment in extensive data collection across diverse ethnic groups and methodological research to enhance genetic prediction in admixed individuals (75). Another critical issue is the interpretability of genetic findings in participants, requiring clinicians to possess the capacity to comprehend and interpret data (76). Furthermore, privacy and security of the involved genetic data must be adequately ensured. Federated learning, as a distributed machine learning technique, aims to achieve collaborative modeling while ensuring data privacy, security, and legal compliance (77). Participants can train their local models using their proprietary data, and through iterative training, each participant contributes to the construction of a global model without sharing their data externally (78). This approach fosters collaboration among multiple medical institutions, facilitating the sharing of model learning outcomes (79).

The likelihood of individuals with undifferentiated arthritis (UA), who exhibit joint symptoms without fulfilling the full diagnostic criteria, subsequently progressing to RA poses a clinical conundrum. Accurate prediction of this progression can facilitate early diagnosis and intervention for those at risk, while concurrently preventing overtreatment and diminishing both the health repercussions and superfluous healthcare expenditures for those unlikely to develop RA (80). Models are increasingly geared towards the evaluation of dynamic variables, reflecting shifts correlated with disease activity, such as gene expression profiles, epigenetic modifications, and a spectrum of detailed symptomatic and clinical markers.

A notable investigation sought to unearth clinically pertinent predictive biomarkers from peripheral blood CD4 T cells in UA patients, employing a support vector machine (SVM) classification model. This approach demonstrated that an integration of the pre-established Leiden predictive rule with a 12-gene risk indicator notably enhanced the prognostic capability from the original (AUC=0.74) to a significantly improved accuracy for seronegative UA patients (AUC=0.84) (29). A comparative analysis of three distinct ML algorithms revealed that a SVM model, which integrated DNA methylation profiles from 40 CpG sites with clinical parameters including disease activity score (DAS) and RF, effectively distinguished individuals with UA who were predisposed to developing RA within one year, achieving an AUC range of 0.85 to 1 (30).

Contemporary studies report promising predictive performance in identifying at-risk individuals within the general population and in forecasting RA development in patients with UA, and that the features having the greatest impact on predictive outcomes were identified and selected as much as possible during model training in order to simplify the model and potentially improve performance and generalizability. More important than performance, however, is the potential for practical clinical application, and future studies will need to examine the generalizability of the model by testing it in populations of multiple ethnicities and regions, and tracking the progression of individuals to RA in larger prospective cohorts to observe the accuracy of the model.

### 3.2 Diagnosis and subtype classification of RA

The diagnostic framework for RA, especially in the context of seronegative RA, is intricate and often obstructed by the absence of potent biomarkers, impeding early detection and management (47). Investigations are thus aimed at the identification of new biomarkers to bridge this gap.

Non-invasive imaging techniques are pivotal in elucidating inflammatory activity and its effects on joint morphology, especially when serological markers are indistinct or inconclusive. These tools are indispensable for both diagnostic purposes and for monitoring treatment efficacy (81). Furthermore, the application of ML algorithms in the analysis of imaging data presents a sophisticated approach to patient classification (82). Üreten K et al. presented a model of a Visual Geometry Group-16 (VGG-16) neural network for hand radiographs augmented by transfer learning to distinguish RA patients from non-RA patients, which achieved an AUC of 0.97 (31). Ultrasound imaging of the metacarpophalangeal joints in RA patients has been categorized for classification purposes, employing a DenseNet-based deep learning model in several regions of interest, significant efficacy was demonstrated in distinguishing between synovial proliferation and healthy and diseased synovium, as evidenced by AUCs exceeding 0.8 (32). Additionally, research has been conducted utilizing hand RGB images and gripforce as features to develop a random forest model with an AUC of 0.97 for distinguishing between individuals with RA and control subjects, thereby offering a supplementary diagnostic tool for RA (33). Image-based predictive models have shown notable performance in research settings, accurately differentiating RA patients from others in various cohorts, thereby contributing to the precision and efficiency of RA diagnosis. These models facilitate the early detection of abnormal changes within the joints, enabling timely intervention and ultimately delaying the progression of RA. However, their clinical application still faces significant challenges. A primary obstacle is the interpretability of the models. Owing to the ‘black box’ nature of deep learning models, the decision-making processes are opaque and difficult to comprehend, which may affect both physician and patient trust and understanding of model predictions (83). To address this limitation, some well-known methods can be utilized: The Class Activation Mapping (CAM) technique helps in understanding the regions of interest within images as attended by the model (84); Shapley Additive exPlanations (SHAP) elucidate the global impact of each feature on the model (85); and Local Interpretable Model-agnostic Explanations (LIME) explicate the local prediction process for individual samples (86). Collectively, these methods provide interpretability tools that enhance comprehension of the model’s decision-making process and improve its interpretability. Future studies are also suggested to involve multi-center collaborations to enhance image collection with the intent to further refine and generalize these diagnostic models.

In RA, both individual analyses and integrative omics studies have accumulated a vast amount of data, providing insights into the mechanisms of RA from multiple perspectives. Genomics identifies genetic variations associated with RA, revealing potential genetic mechanisms influencing gene expression (87). Epigenetic modifications, including DNA methylation, histone modifications, chromatin remodeling, and non-coding RNA, play crucial roles in maintaining normal gene expression patterns. Epigenomics studies these modifications to reveal gene expression and regulatory mechanisms in RA, offering insights into the diverse molecular processes involved (88). Transcriptomics, by analyzing the variations in gene expression under different conditions, provides a detailed elucidation of which genes are upregulated or downregulated in RA. This process not only involves the regulation at the genetic level but also directly affects the production and function of the corresponding proteins (89). Proteomics provides a comprehensive analysis of protein composition, expression levels, and modification states, elucidating the interactions and connections among proteins that may play key roles in RA inflammation and immune response processes (90). Metabolomics provides insights into the shifts in metabolic states and pathways during the progression of RA. These changes are potentially influenced by alterations in gene and protein activities. Furthermore, metabolites themselves can play a modulatory role, affecting gene transcription and protein expression, thereby forming a complex interplay that influences disease dynamics (91). Host genomic variations significantly influence the composition of the gut microbiota, which can synthesize, regulate, or degrade endogenous small molecules or macromolecules, resulting in metabolic changes. Utilizing metagenomics and related techniques reveals the role of gut microbiota in the development of RA by influencing metabolic pathways and modulating the host immune system (92). Omic studies are characterized by the generation of vast, high-dimensional datasets. ML algorithms are critically employed for visualization and processing such information—finding patterns, crafting predictive models, and examining large-scale, multi-omic data to identify biomarkers and pathways implicated in disease progression (93, 94). Existing research has integrated multimodal data and employed various machine learning algorithms to develop high-performance diagnostic models for RA. Key genes highly correlated with RA phenotypes have been identified through the application of weighted gene co-expression network analysis (WGCNA) and differential gene expression (DEG) analysis on RA blood sample microarray datasets. These genes have been deployed as features to assess the performance of six ML models, with five demonstrating commendable efficacy (AUC > 0.85) (34). Through the sourcing of RA patient peripheral blood sample microarray datasets from the GEO database, a platelet-related signature risk score model was formulated, comprised of six genes, using the Least Absolute Shrinkage and Selection Operator (LASSO) algorithm. The model exhibited AUCs of 0.801 and 0.979 across the training and validation sets, respectively (35). Employing the Generalized Matrix Learning Vector Quantization (GMLVQ) method, mRNA expression profiles of cytokines and chemokines from synovial biopsies were analyzed, leading to the identification of two gene sets. These sets were instrumental in generating a model capable of differentiating between various arthritis types, with AUC scores reaching 0.996 and 0.764 for distinguishing diagnosed RA from non-inflammatory cases and early-stage RA from self-remitting arthritis, respectively (36). By focusing on the expression of 19 N6-methyladenosine (m6A) methylation regulators, diagnostic models have been established to separate RA from non-RA conditions. A subset of these regulators, particularly IGF2BP3 and YTHDC2, demonstrated accuracies and AUCs exceeding 0.8 across most ML models, indicating the potential diagnostic importance of m6A methylation profiles (37). A multi-variable classification model, incorporating 26 metabolites and lipids, was devised utilizing three ML algorithms. The logistic regression model, in particular, stood out for its ability to differentiate seropositive and seronegative RA from normal controls within an independent validation cohort, securing an AUC of 0.91, thus showcasing that a holistic metabolomic and lipidomic approach grounded in Liquid Chromatography-Mass Spectrometry (LC-MS) can effectively segregate RA cases (38). Serum antigens were analyzed in patient cohorts with RA, osteoarthritis (OA), and healthy controls. Subsequently, distinct biomarker sets were identified for the differentiation of RA, ACPA-positive RA, and ACPA-negative RA using feature selection through the Random Forest algorithm. The model demonstrated exceptional performance with AUC values of 0.9949, 0.9913, and 1.0, respectively, establishing a proteomics-based diagnostic model for RA (39). Furthermore, leveraging metagenomic data to predict the microbiomic characteristics of the gut in autoimmune diseases has been demonstrated to discriminate between various types of autoimmune disorders (40).

Histopathology, as a fundamental pillar in confirming disease diagnosis, stands as the definitive standard for the verification of numerous ailments (95). Overlap of symptoms in certain pathologies may obscure the principal etiology responsible for articular manifestations; in such instances, tissue biopsy, particularly of synovial tissue, proves invaluable. Following Total Knee Arthroplasty (TKA), synovial samples from 147 OA and 60 RA individuals were subjected to hematoxylin and eosin (H&E) staining. Utilization of a Random Forest Algorithm, integrating pathologist-derived scores with computer vision-generated cellular density measures, led to the construction of an optimal discriminative model for OA and RA, achieving a model AUC of 0.91 (42). This serves as a potent discriminative tool for RA assessment. Orange et al. utilized consensus clustering of gene expression data from synovial tissues of patients with RA to identify three distinct synovial subtypes: high-inflammatory, low-inflammatory, and mixed. They subsequently employed a support vector ML algorithm to distinguish between these subtypes based on histological features, achieving area under the curve values of 0.88, 0.71, and 0.59, respectively (43).

Despite the high performance of ML-derived predictive models for RA diagnosis, concerns on potential model overfitting due to limited sample sizes, which may exaggerate effect sizes, cannot be overlooked. Additionally, independent evaluation of the research methodology, data processing, and outcomes by an external party ensures the accuracy and reliability of the research findings. Validation of these models in diverse datasets, supplemented by molecular biology experimentation, is imperative for evaluating true diagnostic merit. Predictive models relying on histopathological data encounter additional challenges, including the necessity for manual feature annotation by pathologists and the invasiveness of the procedure, compounded by technical and sample handling issues. External validation is a critical quality control measure, ensuring that model utility and accuracy in diagnosing RA reflect true clinical relevance and potential for widespread application. The diagnosis of RA extends beyond segregating RA from healthy subjects or OA patients. Future investigations must address the diagnostic capacity of predictive model-derived markers in distinguishing seronegative RA from other inflammatory arthritides, such as psoriatic arthritis, reactive arthritis, or spondyloarthritis. Concomitantly, safeguarding against confounding variables and maintaining diversity within patient cohorts are essential to render the model universally applicable.

### 3.3 Prediction of disease activity and imaging progression in RA

Radiographic deterioration in RA is characterized by the degree of articular damage and the presence of distinct lesions such as joint space narrowing, bone erosion, and osteoporosis, as revealed through diagnostic imaging modalities including X-rays, magnetic resonance imaging, or computed tomography scans (96). The quantification and prognostication of structural joint impairment traditionally hinge on clinical expertise, underscoring the necessity for an automated, bias-free evaluation method. A study utilizing SVM modeling on cohorts comprising 374 Korean and 399 North American patients with incipient RA identified SNPs correlated with radiographic progression. An integrated model encompassing SNPs with clinical parameters exhibited optimal performance, yielding a mean ten-fold cross-validation AUC of 0.78, providing a more satisfactory distinction between severe and non-severe progression (44).

Radiological damage bears a significant association with disease activity in RA, with heightened activity posing an increased risk for osseous impairment. CNNs trained on ultrasound imagery of RA joints, have facilitated the automatic grading of disease activity, achieving an overall classification accuracy of 83.9% (45). Vodencarevic et al. used data from 135 consultations with 41 RA patients to predict flare incidents during biologic disease-modifying antirheumatic drugs (DMARDs) tapering in remission. They combined multiple ML models to achieve an AUC of 0.81 (46). Furthermore, baseline serum proteomics from 130 stable RA patients in clinical remission was analyzed for biomarkers predictive of future disease flares, employing LASSO and eXtreme Gradient Boosting (XGBoost) algorithms to construct predictive models. The XGBoost model exhibited superior performance in differentiating between relapsed and non-relapsed patients with an AUC of 0.80 (47).

The expansive volume of patient intelligence and clinical information harbored in electronic medical records (EMR) and electronic health records (EHR) constitutes a substantial body of data ripe for investigation (97, 98). Nonetheless, hindrances such as imbalances in data record quantities across patients, omissions of pivotal information, and the variability in patient conditions and therapeutic outcomes over time contribute to the complex temporal nature of the data (48). Conventional ML techniques encounter constraints concerning data pre-processing, time-series analysis capacity, and the simplification of intricate relational processing (99). Deep learning integrated with structured EHR data, have been deployed to prognosticate disease activity during subsequent outpatient rheumatology consultations, wherein the model trained on the UH cohort manifested an AUC of 0.91 for internal validation and 0.74 for external cohort testing (48). Feldman et al. endeavored to enhance the precision of RA disease activity evaluation by integrating electronic medical records and claims data, achieving an AUC of 0.76 in discriminating high/moderate from low disease activity/remission (49). Chandran et al. employed the use of biologic agents or tofacitinib as a surrogate for distinguishing disease severity indicators, with the model accurately predicting both current and future disease activity validated across various databases with AUCs exceeding 0.7 (50).

The aforementioned results substantiate the viability of employing routinely documented clinical and laboratory data to assess and forecast disease activity in RA. With the progressive advancements in information technology, an extensive array of data has become accessible, prompting researchers to explore ML methodologies for the extraction of RA patient records from electronic health record data, thereby enabling the study of substantial populations at minimal expense. Algorithms trained via ML are progressively leveraged with EMR for clinical investigations. These algorithms function by detecting specifiable patterns in the data associated with RA, yet systematic disparities in EMR data quality present hurdles for model generalizability. Despite these challenges, high-caliber investigations are somewhat limited and the dependability and transferability of pertinent ML methods remain largely undetermined, rendering periodic evaluation of algorithm performance imperative. The current research trend involves the utilization of thousands of digitally annotated images obtained from large-scale observational studies, clinical trials, and electronic medical records, along with clinical data, to automatically classify and quantify the extent of joint damage and activity scores in RA using ML algorithms (100–102).

### 3.4 Prediction of RA treatment response

In the realm of RA therapeutics, a plethora of options including nonsteroidal anti-inflammatory drugs (NSAIDs), glucocorticoids, conventional synthetic DMARDs, biologic DMARDs, and oral small molecules have been made available (103). The selection of appropriate treatments continues to challenge clinicians owing to the vast range of alternatives and the prevalent trial-and-error approach in therapeutic prescription, exacerbated by a lack of comprehensive knowledge regarding drug efficacy and safety across distinct patient demographics (53).

Methotrexate (MTX) stands as the quintessential first-line therapy in RA treatment strategies (104). Investigation into whether disparities in the gut microbiome across individuals could serve as predictive markers for MTX efficacy in newly onset RA was conducted by Artacho et al. Fecal samples from 26 new-onset RA patients, procured prior to MTX treatment, were analyzed using 16S ribosomal RNA (16S rRNA) and shotgun sequencing. Subsequent construction of a predictive model via random forests revealed that a response to MTX treatment at 4 months could be anticipated, with an AUC of 0.84, based on colony characterization (51). Additional research involving ML algorithms applied to clinical and biological data from 493 and 239 patients across two cohorts, aimed to predict MTX treatment response at 9 months. Notably, the Light Gradient Boosting Machine (LightGBM) model acquired AUCs of 0.73 and 0.72 in training and external validation sets, respectively (52). Lim et al. analyzed exome sequencing data from 349 RA patients and predicted treatment response to MTX using six ML algorithms. They identified 95 genetic factors and 5 non-genetic factors that influenced response. The predictions had strong performance with AUCs between 0.776 and 0.828 in the test set (53). Plant et al. utilized whole blood samples from RA patients initiating MTX treatment, both before and 4 weeks after commencement, conducting gene expression profiling to foretell treatment response at 6 months. Application of an L2 regularized logistic regression yielded an AUC of 0.78 (54). The development of these predictive models has contributed significantly towards identifying patients who are more likely to respond favorably to, or may not derive benefit from, MTX treatment.

Anti-tumor necrosis factor (anti-TNF) agents have been established as pivotal second-line therapeutic agents following methotrexate. A prospective multicenter study recruited 104 RA patients and 29 healthy donors to discover predictive biomarkers for anti-TNF treatment using ML. A hybrid model combining clinical and molecular variables achieved a high AUC value of 0.91 (55). The DREAM RA Responder Challenge introduced a novel approach to predicting anti-TNF treatment response by proposing an optimal model that incorporates Gaussian Process Regression (GPR) and integrates demographic, clinical, and genetic markers. This model accurately predicts the Disease Activity Score in patients 24 months post-baseline assessment and categorizes treatment response according to the EULAR response criteria, effectively identifying non-responders to anti-TNF therapy with an AUC of 0.6 in cross-validation data (56). Kim et al. utilized 11 datasets containing 256 synovial tissue samples, integrating RA-associated pathway activation scores and four ML types, and found that the SVM model performed the best, with an AUC of 0.87 using the pathway-driven model and an AUC of 0.9 using the DEG-driven model (57).

Recent research has emphasized the potential benefits of integrating diverse datasets for the purpose of treatment decision-making. ML algorithms have demonstrated efficacy in enhancing the precision of response prediction for TNF inhibitors and MTX. Furthermore, ML methodologies are being increasingly utilized in forecasting treatment responses to a range of other biologic therapies (61–64). Clinical data may be limited by trial design, including inclusion and exclusion criteria.Using deep learning technology for cluster analysis on RA patients has revealed the connection between patient characteristics and treatment response (105). Advancements in spatial omics technologies enable a comprehensive and spatially intact analysis of synovial tissue in RA patients. This approach allows for precise localization of cells, exploration of cellular interactions, assessment of cell type distributions, and identification of disease-associated molecular markers (106). Integrating traditional multi-omics with spatial data, spatial multi-omics elucidates the complexity and dynamics of biological processes across various levels, including their interactions and influences on each other. This approach deepens our understanding of the pathological mechanisms of RA and enhances our knowledge of its spatial heterogeneity (107). The biopsy-driven RA randomized clinical trial (R4RA), which utilizes spatial omics to create synovial biopsy gene maps, provides a paradigm for predicting drug treatment responses and refining therapeutic strategies. This is crucial for achieving personalized medicine and optimizing treatment outcomes. Despite some progress, spatial omics in RA research is still in its early stages. Numerous challenges remain, such as high costs, high demands on sample handling, patient acceptance, ethical issues, and the need for advanced computational tools for data integration (108). Overcoming these challenges will be crucial for developing accurate, interpretable, and clinically applicable predictive models. In summary while opportunities exist for refining the accuracy of these predictions, progress is evident in this area of study. In the future, using a larger, more comprehensive datase, appropriate algorithms, and methods in parameter optimization, improving model features and validating against independent cohorts may further improve the discriminative power of predictive models.

### 3.5 Prediction of comorbidities related to RA

ML is also gaining attention in the prediction of comorbidities associated with RA. Focus within extant research has primarily been oriented towards the identification of risk factors for osteoporosis (65, 66), assessment of cardiovascular risk (67, 68), and the prediction of interstitial lung disease development (69) in individuals with RA. Current models pertaining to comorbidities are limited in both quantity and accuracy, with constraints stemming from various sources, notably the scarcity of comprehensive comorbidity data within RA patient cohort datasets. Furthermore, there is significant variability in data quality across different cohorts. To overcome these obstacles, future research should prioritize the accumulation of larger, more robust datasets and improve integration among diverse data sources.Simultaneously, there is a necessity for the advancement of algorithms with broader applicability, thereby enabling the utilization of ML in the prediction of complications associated with RA.

## 4 Conclusion and outlook

Integrating data from diverse sources allows ML models to yield more comprehensive and precise predictions for the diagnosis and treatment outcomes of RA. However, more focus and effort are needed to create predictive models for comorbidities related to RA. Recent research has demonstrated the potential of multimodal learning to improve clinical prediction accuracy. The optimal performing model under specific conditions often necessitates an extensive comparative analysis. Beyond frequently used metrics such as AUC, accuracy, sensitivity, specificity, and F1 score, the employment of cross-validation, the statistical tests applied, the model’s computational cost, the data requirements, and accessibility, the adoption of multimodal learning approaches aims to refine clinical predictions. Efforts should be made to improve the clinical operability of models, utilize external datasets from diverse origins for validation, assess the model’s generalizability, monitor its long-term performance, and evaluate its strengths and weaknesses through multidimensional approaches rather than relying on a single performance metric. Although ML models have demonstrated impressive predictive prowess in research settings, it is imperative to establish their practicality and effectiveness in real-world clinical scenarios. To cultivate trust and acceptance among medical practitioners, it is essential to enhance the interpretability of these models. This can be achieved by prioritizing simplicity in experimental design or by employing tools that enhance model interpretability. Finally, but importantly, the privacy and ethical implications of big biological data should be emphasized and protected.

## Author contributions

YMS: Data curation, Visualization, Writing – original draft. MZ: Data curation, Formal analysis, Writing – review & editing. CC: Data curation, Formal analysis, Writing – review & editing. PJ: Data curation, Formal analysis, Writing – review & editing. KW: Data curation, Formal analysis, Writing – review & editing. JZ: Data curation, Formal analysis, Writing – review & editing. YS: Data curation, Formal analysis, Writing – review & editing. YZ: Data curation, Formal analysis, Writing – review & editing. FZ: Data curation, Formal analysis, Writing – review & editing. XL: Data curation, Formal analysis, Writing – review & editing. SG: Conceptualization, Writing – review & editing. FW: Supervision, Writing – review & editing. DH: Funding acquisition, Supervision, Writing – review & editing.
